# Bacterial Manipulation of Wnt Signaling: A Host-Pathogen Tug-of-Wnt

**DOI:** 10.3389/fimmu.2019.02390

**Published:** 2019-10-17

**Authors:** Madison R. Rogan, LaNisha L. Patterson, Jennifer Y. Wang, Jere W. McBride

**Affiliations:** ^1^Department of Pathology, University of Texas Medical Branch, Galveston, TX, United States; ^2^Department of Microbiology and Immunology, University of Texas Medical Branch, Galveston, TX, United States; ^3^Center for Biodefense and Emerging Infectious Diseases, University of Texas Medical Branch, Galveston, TX, United States; ^4^Institute for Human Infections and Immunity, University of Texas Medical Branch, Galveston, TX, United States

**Keywords:** Wnt, β-catenin, bacteria, pathogen, innate immunity, immunoevasion

## Abstract

The host-pathogen interface is a crucial battleground during bacterial infection in which host defenses are met with an array of bacterial counter-mechanisms whereby the invader aims to make the host environment more favorable to survival and dissemination. Interestingly, the eukaryotic Wnt signaling pathway has emerged as a key player in the host and pathogen tug-of-war. Although studied for decades as a regulator of embryogenesis, stem cell maintenance, bone formation, and organogenesis, Wnt signaling has recently been shown to control processes related to bacterial infection in the human host. Wnt signaling pathways contribute to cell cycle control, cytoskeleton reorganization during phagocytosis and cell migration, autophagy, apoptosis, and a number of inflammation-related events. Unsurprisingly, bacterial pathogens have evolved strategies to manipulate these Wnt-associated processes in order to enhance infection and survival within the human host. In this review, we examine the different ways human bacterial pathogens with distinct host cell tropisms and lifestyles exploit Wnt signaling for infection and address the potential of harnessing Wnt-related mechanisms to combat infectious disease.

## Introduction

The innate immune response is the first, and in many successful cases, the primary barrier between bacterial invader and human host. Fine-tuned through co-evolution with microbial insults and largely evolutionarily conserved across the Metazoa, the innate immune system specializes in both pathogen recognition and the formation of a rapid response (1). At the foundation of the innate cellular antimicrobial response are families of germline-encoded pattern recognition receptors (PRRs) expressed by professional phagocytes, including Toll-like, C-type lectin, NOD-like, and RIG-I-like receptors (2, 3). Interaction of receptor and ligand, which include lipoproteins, polysaccharides, nucleic acids, and other conserved microbial molecular patterns, results in an inflammatory response involving cytokine, and chemokine gene transcription, pathogen clearance through various mechanisms such as lysosomes, antimicrobial peptides, or membrane attack complexes, and coordination of the adaptive immune response (2, 4). Unsurprisingly, bacterial pathogens have evolved an armament of immunoevasion mechanisms as carefully selected for as the immune system that defends the host. The very foundation of bacterial virulence is the ability to subvert host defenses in order to establish a replicative niche. This involves mechanisms beneficial to pathogens that replicate in the extracellular niche, such as serum resistance, tissue adherence, and motility; mechanisms beneficial to the pathogens that replicate in an intracellular niche, such as controlling host cell fate and avoiding lysosomal destruction; and mechanisms employed by pathogens of both niches including competitive nutrient acquisition systems and secretion of effector proteins to modulate the host.

The means by which pathogens subvert the host innate immune responses have helped expand our knowledge regarding how eukaryotic cellular signaling pathways cooperate to modulate innate immunity. Often, these pathways moonlight as branches of the immune system, as they were originally discovered in the context of cell development or cancer. For example, eukaryotic Notch signaling is a well-characterized regulator of cell fate that is highly active in development and tissue homeostasis (5). This pathway has been shown to function within innate immunity through regulation of PRR expression. The obligately intracellular pathogen *Ehrlichia chaffeensis* utilizes a type 1 secretion system (T1SS) effector to activate Notch signaling which indirectly downregulates PU.1, a transcriptional activator of TLR2 and 4 (6). The discovery of xenophagy as a type of autophagy is another example of a moonlighting innate immune pathway. Years of research have demonstrated that a process originally thought only to function as a cellular starvation and stress response also functions within the innate immune system (7). Xenophagy is deployed against Group A *Streptococcus* as a method of bacterial clearance, while other pathogens express effector proteins to escape xenophagy, as is the case with *Listeria monocytogenes* expression of xenophagy evasion protein ActA (8, 9). As evidence linking conserved eukaryotic cell pathways to the immunosubversion of human pathogens continues to grow, as does our model of the innate immune system and the networks that comprise it.

**Figure 1:**
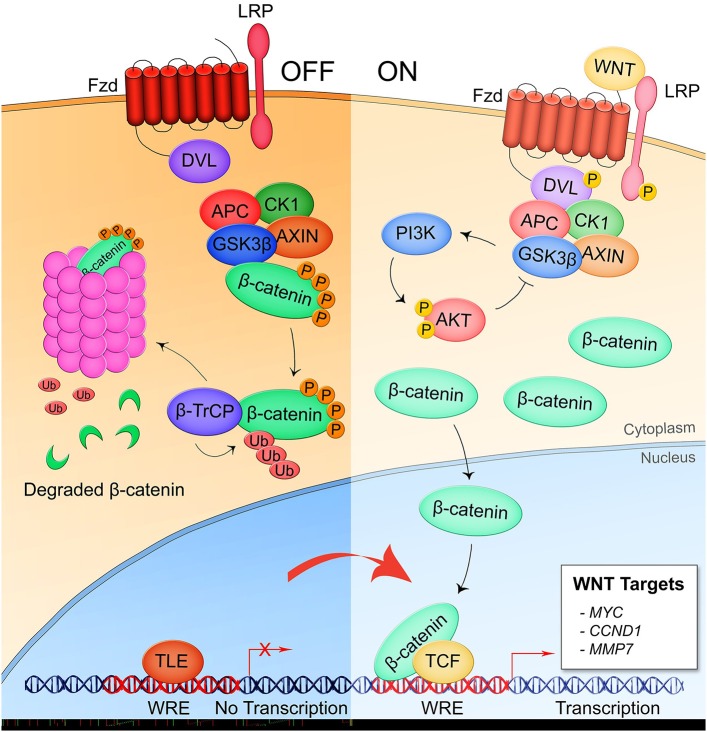
Canonical/β-catenin-dependent Wnt signaling. In the pathway off state, the β-catenin destruction complex consisting of APC, CK1, GSK3β, and AXIN binds β-catenin leading to its phosphorylation by GSK3β and subsequent ubiquitination by the E3 ubiquitin ligase complex β-TrCP which targets β-catenin for proteasomal degradation. Thus, the Wnt response element (WRE) located in the promoter region of Wnt pathway target genes remains bound by transcriptional repressor TLE. When a secreted Wnt ligand originating from the same or a nearby cell binds Fzd and the coreceptor LRP5/6, Dvl is phosphorylated and sequesters the destruction complex, preventing the phosphorylation of β-catenin. Accumulation of β-catenin is the cytoplasm leads to nuclear translocation of the protein where it binds with co-activator TCF at the WRE and drives expression of Wnt target genes.

Mounting research within the last two decades has demonstrated that the conserved eukaryotic signaling pathway Wnt is a significant part of the interplay between the human host and both extracellular and intracellular bacterial pathogens. The discovery of the Wnt pathway began with the identification of the murine oncogene *int-1* that was found to be homologous with a *Drosophila* gene that controlled body segmentation during development (10, 11). Later renamed Wnt proteins, these gene products are a family of 19 highly conserved, secreted, lipidated glycoproteins that regulate metazoan development and tissue homeostasis (12). Wnt proteins participate in paracrine and autocrine signaling through binding of 1 of 10 homologs of the seven-pass transmembrane receptor Frizzled (Fzd1-10) and a cognate coreceptor expressed on the surface of the signal-receiving cell. The signal is transduced through the intracellular mediator Disheveled (Dvl) which, depending on its phosphorylation state, activates either canonical or non-canonical pathways (13). Canonical Wnt signaling, also known as β-catenin-dependent signaling, is the most well-studied Wnt pathway (Figure 1) (14). In the pathway off state, the β-catenin destruction complex consisting of Axin, adenomatous polyposis coli (APC), glycogen synthase kinase 3β (GSK3β), and casein kinase 1 (CK1) facilitates the phosphorylation of β-catenin by GSK3β which induces ubiquitination of β-catenin by the β-TrCP-SCF E3 RING-type ubiquitin ligase complex (β-TrCP) and subsequent proteasomal degradation. When Dvl is activated through interaction of a Wnt ligand with a Fzd receptor and the canonical pathway coreceptor lipoprotein receptor-related protein 5/6 (LRP5/6), the destruction complex is recruited to the Frizzled-Dvl complex at the plasma membrane, freeing β-catenin from degradation. Accumulation of the cytoplasmic pool of β-catenin induces its translocation into the nucleus where it binds with T-cell factor (TCF) transcription factor at the Wnt response element (WRE) DNA sequence and activates transcription of target genes involved in processes such as development (*SNAIL, ENGRAILED, SLUG*) and cell proliferation (*CMYC, CCND1*, and *MMP7*).

**Figure 2:**
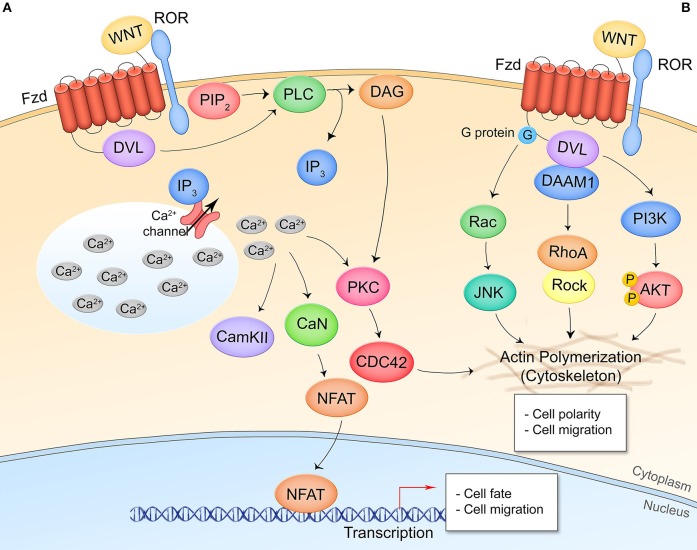
Non-canonical/β-catenin-independent Wnt signaling pathways. **(A)** Wnt/Ca^2+^ signaling. A Wnt ligand interaction with Fzd and the non-canonical coreceptors ROR1/2 activates Dvl which activates PLC, leading to production of IP_3_ and opening of cytosolic Ca^2+^ stores. CAMKII, CaN, and PKC are activated by the increased cytosolic Ca^2+^ levels. CaN dephosphorylates NFAT leading to its nuclear translocation and expression of NFAT target genes. PKC crosstalks with Wnt/PCP signaling. **(B)** Wnt/planar cell polarity signaling. Interaction of Wnt and respective receptor/coreceptor leads to cytoskeletal reorganization through G-protein activation of Ras-JNK signaling, Dvl-DAAM1 association and Rho activation, and Dvl activation of PI3K and Akt. This pathway is transcription-independent.

Non-canonical, β-catenin-independent signaling can be divided into two pathways: the Wnt/Ca^2+^ pathway and the planar cell polarity (Wnt/PCP) pathway. In the Wnt/Ca^2+^ pathway, Wnt ligands signal through Fzd and the coreceptor receptor tyrosine kinase-like orphan receptor 1/2 (ROR1/2) to induce Dvl-dependent phospholipase C (PLC) cleavage of phosphatidylinositol 4,5-biphosphate (PIP_2_), producing inositol triphosphate (IP_3_) and diacyl glycerol (DAG) (Figure 2A) (15). IP_3_ acts on Ca^2+^ channels at the endoplasmic reticulum resulting in a wave of cytosolic Ca^2+^ that drives protein kinase C (PKC) and Ca^2+^/calmodulin-dependent protein kinase II (CAMKII) activity. This controls nuclear translocation of nuclear factor of activated T cells (NFAT) for target gene transcription and actin polymerization through the Rho GTPase CDC42. NFAT target genes have been most thoroughly researched in the context of osteoclast formation and T cell regulation. In the transcription-independent Wnt/PCP pathway, cell polarity and migration are regulated through the direct interaction of Dvl and Dvl-associated activator of morphogenesis (DAAM1), G protein activation of the small GTPase Rac, and Dvl activation of phosphoinositide 3-kinase (PI3K) (Figure 2B) (16). Non-canonical pathways demonstrate a high amount of crosstalk and together regulate events such as filopodia formation, cell movement, and establishment of cell polarity.

Pathogen manipulation of Wnt signaling as a mechanism of innate immune subversion takes advantage of three main outcomes of the signal cascade: cell fate determination (including maintenance of epithelia and endothelia), anti-inflammatory effects, and phagolysosome formation. In this review, we highlight mechanisms of Wnt pathway manipulation by several extracellular, and obligate intracellular pathogens, with a specific focus on the virulence factors involved and the consequences on innate immune subversion. Table 1 summarizes the role of Wnt signaling in the pathogenesis of the bacteria reviewed herein. Ultimately, our understanding of host-pathogen interaction at the cellular and molecular levels will highlight potential targets for therapeutic intervention and expand our model of the many roles of Wnt signaling within the eukaryotic cell.

**Table 1:** Summary of the role of Wnt signaling pathways and respective bacterial factors involved in the pathogenesis of representative bacteria.

**Pathogen**	**Pathway**	**Effect**	**Tissue/cell type**	**Bacterial determinants**	**Mechanism**	**Outcome**	**References**
*Salmonella enterica*	Canonical	Inhibition	Intestinal epithelium	PhoP-PhoQ	Unknown	NF-κB activation; inflammation	(17–19)
			Intestinal capillaries	Spi2	Unknown	Gut-vascular barrier disruption	(20)
		Activation	Crypt-localized epithelial cells	AvrA	β-catenin deubiquitination	Cell proliferation; NF-κB inhibition	(21–23)
			Enterocytes	SopB	β-catenin de-phosphorylation	*RANKL*-mediated epithelial-mesenchymal transition	(24)
*Chlamydia* spp.	Canonical	Activation	Reproductive tract epithelium	Unknown	Adherens junction disruption	Host cell proliferation; *OLFM4* upregulation; chlamydiae development	(25, 26)
			Respiratory epithelium	Cpn1027	Caprin2, GSK3β sequestration	*BCL2*-mediated apoptosis inhibition	(27)
*Rickettsia* spp.	Canonical	Activation	Endothelium	Unknown	Adherens junction disruption; DKK1 inhibition	*IL6, IL8* suppression	(28, 29)
*Ehrlichia chaffeensis*	Canonical	Activation	Monocytes	TRP120, TRP32, TRP47	Direct interaction with pathway components, target genes	Autophagy inhibition	(30–35)
	Wnt/PCP	Activation				Phagocytosis; lysosome biogenesis suppression; mTOR-mediated autophagy inhibition	
	Wnt/Ca^2+^	Activation				Phagocytosis	
*Mycobacterium tuberculosis*	Canonical	Activation	Macrophages	Unknown	Wnt3a-Fzd1 signaling	Pro-inflammatory cytokine suppression	(36–38)
	Wnt/Ca^2+^	Activation		Unknown	Wnt5a-dependent PIAS1 and SOCS1 expression; Ca^2+^-regulation of phagocytosis	Inhibition of TLR signaling; inhibition of phagosome-lysosome fusion	(39, 40)
	Non-canonical	Activation		Unknown	Wnt6-G protein-ERK-induced *MYC* expression	Anti-inflammatory M2 macrophage phenotype	(41)
*Clostridium difficile*	Canonical	Inhibition	Colonic epithelium	TcdA	Inhibition of Rac1-mediated β-catenin nuclear transport; β-catenin degradation	Suppression of cell proliferation	(42)
				TcdB	Fzd binding	Intestinal epithelium weakening; inflammation	(43, 44)
*Helicobacter pylori*	Canonical	Activation	Gastric epithelium	CagA; T4SS (CagA-independent)	E-cadherin cleavage; methylation of Wnt antagonist genes	Cell proliferation; intestinal transdifferentiation	(45, 46)
	Wnt/Ca^2+^	Activation			Unknown	Intestinal transdifferentiation	(47)
*Pseudomonas aeruginosa*	Canonical	Inhibition	Intestinal epithelium	PAI	Adherens junction disruption	Epithelium weakening	(48, 49)
			Lung epithelium	LecB	β-catenin degradation	NF-κB activation; cell cycle arrest; delayed tissue recovery	
*Escherichia coli*	Wnt/Ca^2+^	Inhibition	Bladder epithelium	Unknown	Wnt5a suppression	Cell Differentiation	(50–52, 153)
	Wnt/Ca^2+^	Activation		Unknown	EZH2-mediated Wnt5a expression	Cell proliferation	
	Canonical	Activation	Intestinal epithelium	Unknown	EZH2-mediated WIF1 repression	Crypt hyperplasia	
			Bladder epithelium	HlyA	β-catenin degradation	NF-κB inhibition; immunosuppression	

## Intracellular Pathogens

### Salmonella enterica

The Gram-negative, facultative intracellular bacillus *Salmonella enterica* causes typhoid fever or non-typhoidal salmonellosis in humans. The bacteria are commonly foodborne pathogens but can also be transmitted fecal-orally (53). *Salmonella* establish infection in the gut where they replicate within the lumen until sufficient numbers induce their entry into M cells which is triggered by T3SS effectors. *S. enterica* are also phagocytosed by various phagocytic cells but can survive phagolysosome acidification and replicate within the intracellular vacuole. Dissemination to other organs is accompanied by a robust immune response and the potential for persistent infection within various cell types. Crossing of the gut barrier and infection of infiltrating immune cells including neutrophils, monocytes, and macrophages are essential to dissemination.

**Figure 3:**
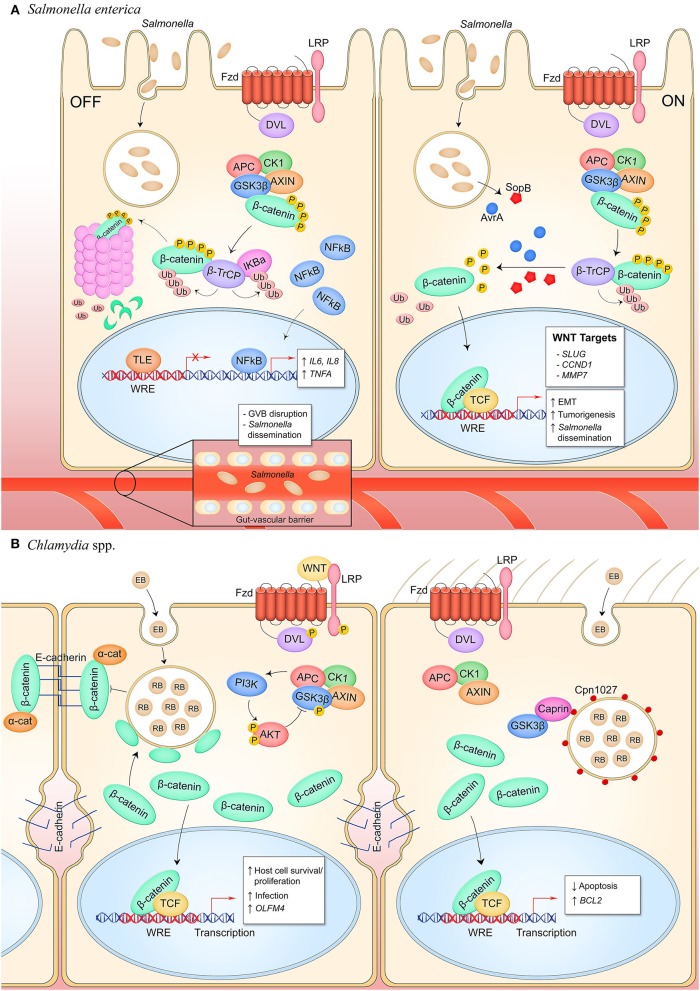
Canonical Wnt signaling manipulation during bacterial infection of epithelial cells. **(A)** *S. enterica* infection of intestinal epithelial cells inhibits Wnt signaling through an unknown mechanism (left). Activity of the E3 ubiquitin ligase β-TrCP, which targets both β-catenin and IκBα for proteasomal degradation, stabilizes NF-κB levels which causes nuclear translocation of the protein and expression of pro-inflammatory target genes. Wnt signaling is also inhibited in the GVB during infection, promoting bacterial access to vasculature by increasing vascular permeability. *S. enterica* secretes the T3SS effectors AvrA and SopB into intestinal M cells and crypt-localized epithelial cells which causes activation of canonical Wnt signaling (right). Deubiquitinase AvrA and phosphatase SopB induce pathway activation through reversal of β-catenin posttranslational modifications, promoting expression of β-catenin-dependent genes that drive EMT in M cells, induce intestinal stem cell proliferation, and inhibit NF-κB activity in stem cells. **(B)** *C. trachomatis* infection of reproductive tract epithelium induces breakdown of adherens junctions and accumulation of β-catenin in the cytoplasm (left). β-catenin localizes to the chlamydial inclusion and translocates into the nucleus to activate transcription. Pathway activity promotes the bacterial developmental cycle through unknown mechanisms as well as drives expression of *OLFM4* which is known to inhibit NF-κB signaling. *C. pneumoniae* expresses inclusion membrane protein Cpn1027 during infection of respiratory epithelium (right). Cpn1027 recruits Caprin2, a scaffold protein of the β-catenin destruction complex, as well as GSK3β, thereby reducing β-catenin turnover and allowing nuclear translocation for expression of target gene *BCL2* to inhibit host cell apoptosis.

Nearly two decades of research have created the model of *Salmonella enterica* serovar Typhimurium manipulation of canonical Wnt signaling to promote infection (Figure 3A). Curiously, *S*. Typhimurium suppresses Wnt signaling in intestinal epithelium and the underlying capillary endothelium but activates the pathway in intestinal stem cells in what appears to be a cell type- or temporally-specific mechanism. In transformed T84 colon carcinoma cells, wild type *S*. Typhimurium represses pathway activation as identified by significant suppression of β-catenin levels that correlates with decreased formation of the TCF-β-catenin complex in the nucleus, dampened expression of the Wnt target gene *CMYC*, and suppression of cellular proliferation (17). This is contrasted with colonization by non-pathogenic *S*. Typhimurium strain PhoP^C^ which possesses an attenuating, constitutively active PhoP-PhoQ two-component system and does not suppress β-catenin-TCF complex formation, implicating a role for this response regulator in suppression of Wnt signaling by pathogenic salmonellae in the colonic epithelium, both in cell culture and in a mouse model (18).

The effects of *S*. Typhimurium suppression of Wnt signaling in the intestines have created two models for bacterial host manipulation by Wnt signaling modulation. First, *Salmonella* studies have revealed a mechanism behind the immunosuppressive effects of β-catenin signaling. IκBα negatively regulates NF-κB transcriptional activity through cytosolic sequestration of NF-κB. It has been shown that β-catenin associates with this complex, indirectly stabilizing IκBα through an unknown mechanism (19). Activation of the NF-κB pathway causes ubiquitination of IκBα by the ubiquitin E3 ligase β-TrCP, the same ligase that targets β-catenin for degradation to suppress Wnt pathway activity, which results in ubiquitin-dependent degradation of IκBα (54). Activation of this E3 ligase therefore results in silencing of Wnt signaling through loss of β-catenin and activation of NF-κB signaling through loss of IκBα repression, while suppression of β-TrCP has the opposite effect. During *S*. Typhimurium infection of colonic epithelial cells, the NF-κB target genes *IL6, IL8*, and *TNFA* are induced, corresponding with enhanced ubiquitination of IκBα as well as β-catenin degradation which demonstrates the reciprocal activation of these two pathways by infection (18, 19). Infection in the presence of lithium chloride, a β-catenin stabilizing agent, or a constitutively active β-catenin mutant *CTNNB1*^−/Δ45^ attenuates this effect. Activation of NF- κB signaling through direct inhibition of β-catenin-dependent Wnt signaling induces the proinflammatory response which recruits dendritic cells and macrophages. While TNF-α is important for controlling replication and spread of bacteria, the cells producing it are also host cells for both replicative and persistent salmonellae, thereby supporting survival and dissemination of the bacteria (53, 55, 56).

Recently, another pathogenic mechanism was discovered by which salmonellae inhibit canonical Wnt signaling at the gut-vascular barrier (GVB), a system of tight and adherens junctions in the capillaries underlying the gut epithelium that functions as a size-selective barrier to molecules traversing the gut barrier (20). Similar to the blood-brain barrier, the GVB is regulated by canonical Wnt signaling (20, 57). This protective barrier is impermeable to bacteria, but infection by *S*. Typhimurium results in downregulation of Wnt signaling in the endothelium which enhances vascular leakiness and promotes bacterial dissemination (20). In the presence of recombinant canonical pathway ligand Wnt3a, canonical signaling is activated and *S*. Typhimurium-induced leakiness is reduced. *S*. Typhimurium possess two pathogenicity islands (Spi1 and Spi2) that together encode the T3SS. A strain lacking Spi2 is unable to induce vascular permeability, indicating this pathogenic mechanism is Spi2-dependent. This model demonstrates how salmonellae permeabilize endothelial layers utilizing T3SS factors without directly manipulating cell-cell contacts, a mechanism that is employed by other endothelial pathogens such as *Rickettsia*. Whether this implicates activation of Wnt signaling as a potential therapeutic for *S. enterica* infection, and whether specific *S. enterica* pathogenicity island effectors are responsible for Wnt pathway inhibition remains to be investigated.

Studies using a *Salmonella* colitis mouse model have demonstrated that *S*. Typhimurium is able to stimulate Wnt signaling in intestinal stem cells in contrast to the inhibitory mechanism deployed in the epithelium and endothelium. The T3SS effector AvrA, a deubiquitinase, has been shown to activate canonical Wnt signaling in intestinal stem cells through deubiquitination and subsequent stabilization of β-catenin (21, 58). Additionally, AvrA alone, in the absence of infection, can activate pathway activity. In the colon of the *Salmonella* colitis mouse model, stem cells demonstrate β-catenin nuclear localization and expression of Wnt target genes *MMP7* and *CCND1* (22). Hyperactivation mediates pathological effects, and studies have shown that in mice with induced inflammation, infection with AvrA^+^ *S*. Typhimurium drives tumorigenesis (22). In addition to AvrA, T3SS effector SopB has tumorigenic properties through inducing cellular transformation of follicular-associated epithelial enterocytes into microfold cells (24). This has also been linked to a canonical Wnt-dependent mechanism. The phosphatase dephosphorylates β-catenin and Akt in primary rectal epithelial cells, resulting in β-catenin-dependent signaling and expression of target gene *SLUG* which activates RANKL expression, a critical cytokine for M cell development (24, 59). Indeed, increased M cells is a phenotype of *S. enterica* infection and proliferation of this cell type promotes *S*. Typhimurium invasion of the intestines for enhanced survival and dissemination.

*S*. Typhimurium has proven to be a model pathogen for understanding the role of canonical Wnt signaling in both gut tissue maintenance and suppression of inflammatory pathway signaling, physiological processes that are applicable to a range of pathogens that occupy a similar niche. Insight to *Salmonella* inhibition of signaling to perturb the GVB brings to light the therapeutic potential of Wnt ligands to maintain endothelial barriers. R-spondin3, a Wnt homolog, has been shown to exhibit an anti-inflammatory effect in an ischemia/reperfusion mouse model and induces tightening of endothelial junctions and loss of vascular leakiness (60). Furthermore, understanding how effectors SopB and AvrA contribute to pathway manipulation during infection highlights the potential of such mechanisms as therapeutics for infection or other disease states mediated by dysregulated Wnt signaling.

### *Chlamydia* spp

*Chlamydia trachomatis* and *Chlamydia pneumoniae* are obligately intracellular, anaerobic pathogens that typically target the human genital and respiratory tract mucosal epithelial cells, respectively. Chlamydiae undergo a developmental cycle in which they transition between two ultrastructural forms at different phases of infection (61). The elementary body (EB) is the infectious form of the bacteria that triggers entry into the host cell. Once intracellular, the chlamydiae transition into the replicative reticulate body (RB) form within the inclusion, the membrane-bound microcolony. The intracellular chlamydiae interact with the host cell through both T3SS effector proteins as well as inclusion membrane-localized proteins that interface with the host cell cytoplasm. Environmental cues such as drug presence or immunological stress can induce the EB to enter a persistent form in which they evade immune detection by entering a dormant state within the inclusion (62). At the end of the infection cycle, the RB transition back into and EB and leave the cell through lysis or exocytosis to infect a neighboring cell.

*C. trachomatis* targets epithelial cells of the genital tract, including the endometrial and fallopian epithelium. Wnt/β-catenin signaling is known to maintain epithelial cell homeostasis through regulating tissue renewal and cell proliferation, and through facilitating epithelial barrier integrity by the β-catenin-E-cadherin complex that constitutes adherens junctions (63–65). *C. trachomatis* infection in the fallopian tube has been shown to disrupt adherens junctions and cause redistribution of β-catenin from the plasma membrane to the chlamydial inclusion (25) (Figure 3B). It is unclear if disruption of these junctions amplifies β-catenin nuclear localization and target gene expression, but infected epithelium does demonstrate increased Wnt pathway activity evidenced by phosphorylation-dependent inactivation of GSK3β and redistribution of APC which indicates inactivation of the β-catenin destruction complex (25). Additionally, inhibition of Wnt signaling through either RNA silencing of β-catenin or a small molecule inhibitor reduces infectivity of the chlamydiae and impairs chlamydiae intracellular development (25, 26). Thus, signaling is beneficial to chlamydiae and may be synergistically activated through inhibition of the β-catenin destruction complex and disruption of adherens junctions. The known beneficial phenotypes of Wnt signaling for the chlamydial niche are 2-fold. First, infection causes Wnt signaling-dependent host cell proliferation, a critical survival strategy for an obligately intracellular pathogen that can maintain persistent infection (25). Second, Wnt signaling upregulates the stem cell marker OLFM4 which is a suppressor of NOD1/2 and NF-κB-dependent pro-inflammatory cytokine expression (23). Therefore, Wnt signaling appears to be an active mechanism of pathogenesis by which *Chlamydia* establishes infection and suppresses NF-kB-mediated innate immune mechanisms.

*C. pneumoniae* inclusion protein Cpn1027 is the only chlamydial protein known to directly interface with the canonical Wnt pathway. During *C. pneumoniae* infection of respiratory epithelium, Cpn1027 directly binds the Caprin2, an adaptor protein within the β-catenin destruction complex (27, 66). GSK3β also localizes to the Cpn1027-Caprin2 complex and demonstrates decreased kinase activity. Consequently, β-catenin translocates to the nucleus and drives expression of the anti-apoptotic *BCL2* gene, linking the infection phenotype of apoptosis inhibition to manipulation of Wnt signaling for enhanced intracellular survival. This mechanism of pathway manipulation is unique to *C. pneumoniae* as Cpn1027 is not expressed by other species of the *Chlamydia* genus (67).

Several questions remain regarding the role of Wnt signaling during *Chlamydia* spp. infection. The consequence of β-catenin localization to the *C. trachomatis* inclusion concurrent with adherens junction disruption is unclear, as signaling is not inhibited in the host cell, and β-catenin is necessary for the chlamydial intracellular life cycle. The chlamydial deubiquitinase ChlaDub 1 has been shown to deubiquitinate NF-κB inhibitor IκBα in order to suppress the NF-κB-dependent expression of proinflammatory cytokines (68). β-catenin and IκBα are both substrates of the E3 ligase SCF^β−*TrCP*^, raising the question of whether β-catenin localizes to the chlamydial inclusion to also serve as a ChlaDub 1 substrate which would lead to pathway activation (69). Identifying interactions between chlamydial secreted effectors or inclusion proteins and components of the Wnt pathway will define novel host-bacterial pathogenic interactions that can be targeted by therapeutics. In addition to understanding pathogenic mechanisms, further research will shed light on chlamydial mechanisms of cellular transformation. *Chlamydia trachomatis* is associated with cancer development through infection-induced degradation of p53 and dysregulated ROS production (70–72). It is well-known that canonical Wnt signaling is a driver of tumorigenesis in multiple human cancers, including cervical cancer (73). However, a model for cervical cancer development through *C. trachomatis*-induced hyperactivation of Wnt signaling has not been investigated.

### *Rickettsia* spp

The *Rickettsia* genus comprises 27 species of obligately intracellular Gram-negative bacteria, over half of which are human pathogens primarily transmitted by various arthropod vectors (74). *R. conorii* and *R. rickettsii* are both members of the spotted fever group of *Rickettsia* and causative agents of the human diseases Mediterranean spotted fever and Rocky Mountain spotted fever, respectively. These pathogens establish infection in the endothelium which induces an inflammatory response consisting of increased vascular permeability, recruitment and activation of natural killer cells and macrophages, and ROS- and cytokine-mediated vascular damage. The bacteria escape from their endocytic vesicle and replicate in the cytosol where they utilize actin-based motility for cell-to-cell spread. Robust production of cytokines IL-6 and IL-8 correlate with infection lethality, and clearance of the bacteria is typically mediated by PRR engagement (75).

Wnt signaling has a complex role in the endothelium, and activation of the pathway induces endothelial cell proliferation and enhanced interaction between endothelial cells and monocytes (76, 77). Additionally, β-catenin is present at endothelial adherens junctions, regulating cell-cell contacts (78). The Wnt signaling pathway controls neovascularization during development but demonstrates decreased activity in adult vasculature (79). However, activation in certain disease states including infection and cancer can induce Wnt-dependent vascular endothelial growth factor A (VEGF-A) expression followed by angiogenesis (76). Activated endothelial cells also release DKK1, a member of the Dickkopf family of secreted Wnt signaling antagonists that exert their effect by outcompeting Wnt ligands for binding of coreceptor LRP5/6 (80). DKK1 is a β-catenin target gene that functions through feedback inhibition of Wnt signaling, thereby decreasing neovascularization induced by VEGF-A (81, 82).

**Figure 4:**
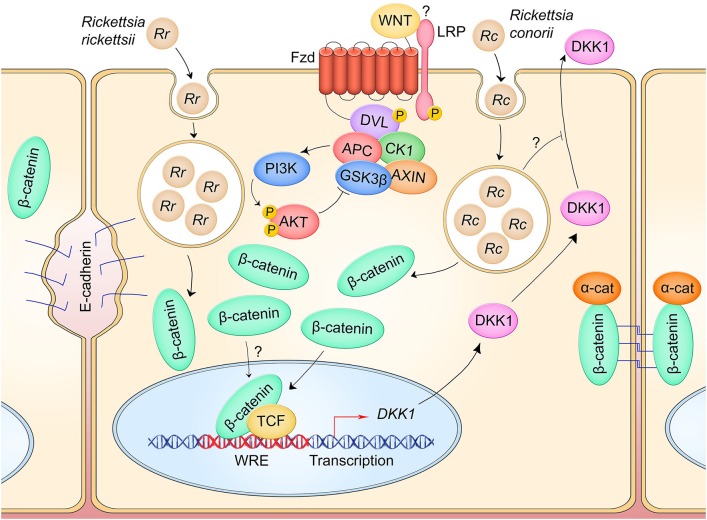
Canonical Wnt signaling manipulation during *Rickettsia* spp. infection of endothelium. *R. rickettsii* (Rr) infection of endothelial cells induces breakdown of adherens junctions and accumulation of cytoplasmic β-catenin which may drive pathway activity (left). *R. conorii* (Rc) infection leads to a suppression of secreted Wnt antagonist DKK1, which may facilitate activation of Wnt signaling to induce an anti-inflammatory environment during infection. IL-6 and IL-8 secretion are suppressed during infection in a DKK1-dependent manner.

In a HUVEC model of *R. conorii* infection, β-catenin rapidly localizes to the nucleus within 2 hpi, indicating activation of Wnt signaling early in infection (Figure 4) (28). It is unclear how the pathway is activated by rickettsiae, as other markers of signaling activity have not been investigated. In a HUVEC model of *R. rickettsii* infection, adherens junctions are disrupted and β-catenin is redistributed from primarily membrane-localized to diffuse localization throughout the cell (29). A correlation was identified between vascular permeability through loss of adherens junction integrity as well as increased expression of NF-κB-dependent inflammatory cytokines, but whether this is related to canonical Wnt signaling reciprocal regulation of the NF-κB signaling pathway is unknown. Increased nuclear entry of β-catenin during rickettsial infections has not been specifically investigated, but consistent with other models of infection-induced adherens junctions remodeling, it is likely that *Rickettsia*-induced cell contact disruption can activate canonical Wnt signaling through increasing the cytoplasmic pool of β-catenin.

In accordance with Wnt signaling activation, DKK1 secreted protein level is significantly reduced in a HUVEC model of *R. conorii* infection relative to uninfected controls from 5 to 180 hpi (28). Infection activates endothelial cells and causes significant increase in secreted protein levels of inflammatory cytokines IL-6 and IL-8. However, RNA-mediated silencing of DKK1 in infected HUVECs significantly reduces production of these cytokines, suggesting that DKK1 plays a role in modulating the inflammatory response. It is hypothesized that *R. conorii* suppresses DKK1 to prevent negative regulation of Wnt signaling, thereby keeping proinflammatory cytokine expression relatively low during infection.

These studies highlight a unique role of Wnt signaling feedback inhibitors in pathogenic mechanisms of infection. In seeking to augment Wnt signaling for its anti-inflammatory effect, *Rickettsia* may possess active mechanisms for reducing the activity of Wnt pathway antagonists like DKK1. A critical gap in this model is whether DKK1 is inhibited at the transcriptional, translational, or post-translation level. Evidence suggests *Rickettsia* may be inhibiting DKK secretion, although further studies are needed to confirm this. This research also presents the therapeutic potential of harnessing the Wnt pathway to mitigate disease. Recombinant DKK1 has been shown to reduce pathological Wnt signaling activation in a mouse model of neovascularization-induced blindness (81). DKK1 levels in *R. conorii*-infected patient sera indicate a decline in secreted DKK1 throughout infection, but whether this is a direct result of pathogen manipulation has not been investigated (28). Altogether, these results propose a complex model in which intracellular pathogens act on feedback inhibition mechanisms of Wnt signaling to interfere with pathway activity, and they propose a unique role for DKK1 in influencing the proinflammatory response to *Rickettsia*.

### Ehrlichia chaffeensis

*Ehrlichia chaffeensis* is the causative agent of the tick-transmitted disease human monocytic ehrlichiosis (HME). The pathogen is an obligately intracellular bacterium and infects mononuclear phagocytes including monocytes. *E. chaffeensis* enters the host cell through phagocytosis and replicates within a membrane-bound vacuole to form a microcolony known as a morula (83). The morula resembles an autophagosome, but through pathogenic mechanisms never fuses with the lysosome (31). Similar tochlamydiae, ehrlichiae undergo a biphasic developmental cycle within the host cell (84). The infectious dense-cored cell (DC) ehrlichiae invade the host cell and transition into replicating reticulate cells (RC) that divide through binary fission. The ehrlichiae complete their infection cycle by transitioning back into DC, rupturing the host cell, and spreading hematogenously to the next host cell. Throughout infection, ehrlichiae secrete a variety of T1SS and T4SS effectors, including the TISS tandem repeat protein (TRP) effectors TRP32, TRP47, and TRP120 that induce pathogenesis through direct interactions with numerous host proteins and host DNA (34, 35, 85, 86). Through unknown mechanisms, the TRPs also localize to the surface of the ehrlichiae and decorate the outer membrane, facilitating interactions with the host cell leading to invasion (87, 88).

**Figure 5:**
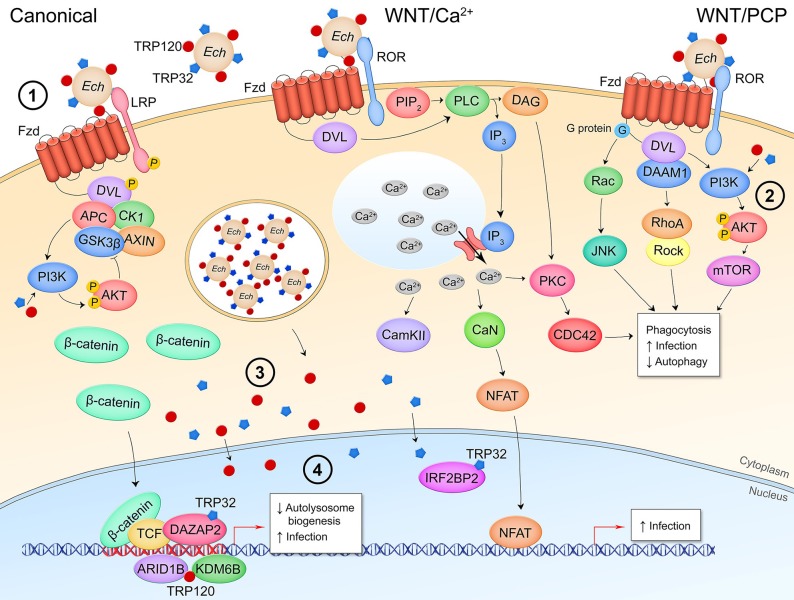
Canonical and non-canonical Wnt signaling manipulation during *E. chaffeensis* infection of monocytes. (1) *E. chaffeensis* dense-cored cells express surface proteins TRP32 and TRP120 that stimulate phagocytosis in a Wnt signaling-dependent manner, potentially through interaction with the Wnt receptor complex and stimulation of non-canonical Wnt pathways to induce cytosolic Ca^2+^ flux and cytoskeletal reorganization. (2) Pathway activity during bacterial entry as well as T1SS secreted effectors TRP32 and TRP120 direct stimulation of PI3K-AKT signaling facilitates mTOR activation which prevents fusion of the bacterial replicative autophagosome with the lysosome. During infection, the secreted TRP effectors localize to the host cytoplasm where they interact with proteins involved in Wnt signaling (3) as well as translocate to the nucleus where they bind host DNA within the promoter regions of Wnt target genes or directly interact with transcriptional regulators of Wnt target genes (4).

*E. chaffeensis* has emerged as a model organism for pathogenesis mediated by the hijacking of conserved cell signaling pathways including Wnt signaling (87). Both canonical and non-canonical Wnt signaling are active early during infection and are necessary for enhancement of ehrlichial infection, as inhibitors or gene silencing of canonical and non-canonical pathway components significantly reduce *E. chaffeensis* survival within the monocyte (Figure 5) (30, 32). Of note, RNA silencing of antagonist DKK3 results in increased infection, while RNA silencing of canonical and non-canonical pathway components such as CK1, CAMKII, NFAT, and β-catenin significantly reduces infection. *E. chaffeensis* enters the host cell via phagocytic pathways regulated by Ca^2+^ signaling and actin filamentation, and the morula labels with autophagosomal markers LC3 and beclin-1 even though autophagy appears to be inhibited and lysosomal marker LAMP2 never localizes to the morula (31, 89, 90). The Wnt pathway has been shown to involved in *E. chaffeensis* phagocytosis because microspheres coated in ehrlichial surface TRPs can stimulate phagocytosis by monocytes but are unable to do so in the presence of a small molecule inhibitor of Wnt signaling (30). Additionally, RNA silencing of Wnt receptors Fzd5 and Fzd9, and Wnt coreceptor LRP6, significantly reduces the number of intracellular bacteria, indicating a potential role for Fzds as *E. chaffeensis* receptors. The hypothesis that *E. chaffeensis* uses activation of non-canonical Wnt signaling to drive actin filamentation and bacterial uptake is supported by evidence demonstrating Wnt5-Fzd5-PI3K non-canonical Wnt signaling induces uptake of non-pathogenic *E. coli* without leading to bacterial killing, as well as the role of non-canonical Wnt signaling in cytoskeletal control (91–94). *E. chaffeensis* avoidance of lysosomal fusion was also found to be dependent on ehrlichial activation of Wnt-PI3K signaling, mediated by TRP32 and TRP120, to stimulate mTOR inhibition of autophagy as well as inhibition of TFEB-dependent lysosome synthesis genes (31). Accordingly, inhibition of Wnt signaling following *E. chaffeensis* infection in monocytes promotes colocalization of lysosomal marker LAMP2 with the ehrlichial cytoplasmic vacuole (31). β-catenin activation is known to repress the autophagy protein p62, indicating another potential mechanism by which ehrlichial activation of canonical Wnt signaling drives intracellular survival (95).

In addition to manipulation of Wnt pathway activation early during infection to facilitate phagocytosis and intracellular survival, *E. chaffeensis* maintains pathway activity throughout infection of the monocyte (30). Yeast-two-hybrid data identified multiple interactions between TRP32 or TRP120 and Wnt signaling pathway components and regulators in the host cytoplasm and nucleus, implicating intracellular pathway manipulation through direct interference with Wnt signaling regulators (85, 86). These interacting partners include pathway negative regulators CEP164 (96), KLHL12 (97), ILF3 (98), and LMO2 (99); and positive regulators PPP3R1 (100) and VPS29 (101). RNA-mediated knockdown of these interacting targets results in significant, differential regulation of THP-1 monocyte infection which demonstrates these TRP-host protein interactions are relevant to *E. chaffeensis* establishment of infection (32). TRPs also interact with nuclear proteins involved in Wnt target gene epigenetic modification and expression, including the Wnt response element transcriptional repressor TLE4 (102), co-activator DAZAP2 (103), and histone remodeling proteins ARID1B, KDM6B, and IRF2BP2 (104–106). Furthermore, TRP32, TRP47, and TRP120 DNA-binding motifs are within the promoter region of numerous Wnt target genes, indicating the TRPs may directly influence Wnt gene transcription through nucleomodulin activity (33–35). Although these specific interactions have not yet been investigated, it is likely that the nucleomodulin activity of TRP effectors or the recently discovered ubiquitin ligase activity of TRP120, plays a role in manipulation of these target proteins, facilitating Wnt pathway activity and ultimately enhancing *E. chaffeensis* intracellular survival (107).

*E. chaffeensis* stands out among human pathogens as a bacterium that targets Wnt signaling at both canonical and non-canonical levels and takes advantage of a wide range of pathway outcomes. Gaps that remain to be filled in the *E. chaffeensis* monocyte infection model include how both signaling branches are activated. The studies reviewed demonstrate that *E. chaffeensis* utilizes an extracellular mechanism of pathway activation, potentially through surface TRPs interaction with the Wnt receptor complex. While many bacteria use secreted effectors to activate Wnt signaling through manipulation of midstream signaling components, ehrlichial TRPs are the only bacterial factors that are known to trigger entry into a cell in a Wnt-dependent mechanism which suggests the surface proteins mimic Wnt ligands and utilize Wnt control of the cytoskeleton for phagocytosis. *E. chaffeensis* demonstrates a potential for intracellular activation of signaling through the secretion of TRP effectors that interact with Wnt pathway-related proteins and DNA in the cytosol and nucleus. These dual modes of extra/intracellular pathway activation may be necessary for temporal regulation of signal activity to facilitate the bacterium's complex intracellular developmental cycle. Understanding the relevance of these vast interactions will shed light ehrlichial pathogenesis while also serving as a model for other intracellular bacteria that reprogram the host cell to establish a replicative niche.

### Mycobacterium tuberculosis

The causative agent of one of the most prevalent infectious diseases in the world, *Mycobacterium tuberculosis* establishes infection in the lower respiratory tract and infects alveolar macrophages as well as epithelial cells. The outcome of infection involves a delicate balance between the innate immune response control of intracellular bacterial killing early in infection and containment of infection by the adaptive immune response in lung granulomas to prevent dissemination. However, this can result in persistent infection with the ability to transition into an acute infection even decades after the initial exposure.

Canonical Wnt signaling in infected macrophages in the lung has been shown to be part of the innate immune response to *M. tuberculosis* and is regulated by the Wnt3a-Fzd1 signaling axis, with significant crosstalk from other Wnt ligands as well as TLR signaling. Although canonical signaling is involved in activation of macrophages, it has also been shown to lead to suppression of pro-inflammatory cytokine expression (36–38). It is hypothesized this anti-inflammatory effect contributes to tissue renewal following activation of the inflammatory response, but canonical pathway activation prior to complete clearance of the bacteria can also facilitate persistent infection. Several non-canonical pathways also play a part in *M. tuberculosis* infection, including Wnt/Ca^2+^ signaling which may be responsible for Ca^2+^ signaling that is necessary for recruitment of phagosome coat protein TACO which allows the bacterium to avoid the lysosomal pathway (39). Wnt5a signaling drives expression of PIAS1 and SOCS1 which inhibit TLR signaling, thereby controlling innate immune coordination (40). A non-canonical signaling pathway in which Wnt6 signals through G proteins to activate ERK and drive *MYC* expression has also been identified as a mechanism of induction of an anti-inflammatory M2 macrophage phenotype which may cooperate with canonical Wnt signaling suppression of inflammation to not only permit tissue renewal but also allow pathogen persistence.

Wnt signaling plays a large role in the processes that both permit and control mycobacterium survival. For further detail, the authors would like to direct the reader to a publication from 2017 that thoroughly reviews the mechanisms controlled by various Wnt ligands during *M. tuberculosis* infection (108).

## Extracellular Pathogens

### Clostridium difficile

The anaerobic, Gram-positive bacterium *Clostridium difficile* is part of the human intestinal normal flora. It is the causative agent of pseudomembranous colitis (PMC), a severe inflammatory disease of the colon that arises from opportunistic infection by *C. difficile* typically following antibiotic-induced disturbances in the normal flora population proportions (109). The bacteria are transmitted fecal-orally in the form of spores and upon environmental triggers, such as exposure to bile acids, enter a vegetative state in the intestines. *C. difficile* pathogenesis is highly dependent on the expression of a family of exotoxins that includes toxin A (TcdA) and toxin B (TcdB) (110). The toxins are secreted from *C. difficile* through non-canonical secretion mechanisms and are endocytosed by target intestinal epithelial cells (111). These toxins are glucosyltransferases that glucosylate host small GTPases including Rho and Ras. Initially, TcdA, and TcdB together were thought to be responsible for symptoms of PMC. Alone, TcdA, but not TcdB, in small animal models can induce clinical signs of PMC including tissue damage, intestinal endothelial leakage, and inflammation (112). However, TcdA^−^/TcdB^+^ clinically relevant strains have been isolated, demonstrating TcdB is sufficient for induction of PMC in humans (113).

**Figure 6:**
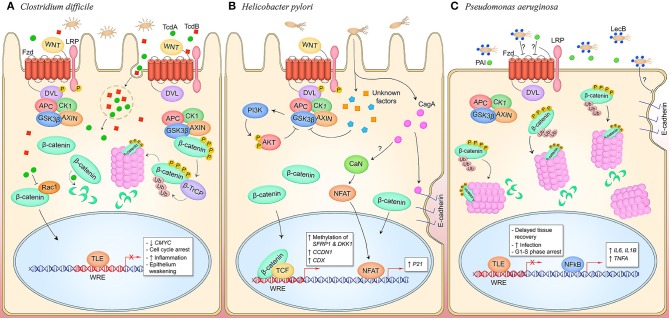
Canonical and non-canonical Wnt signaling manipulation by pathogens occupying an extracellular niche. **(A)** *C. difficile* secretes toxins TcdA and TcdB which are phagocytosed by host cells in the intestinal and colonic epithelia. TcdA glucosyltransferase activity inhibits small GTPases like RacA which may inhibit pathway activity through inhibiting nuclear translocation of β-catenin. TcdB interacts with Wnt pathway components LRP6, Wnt5a, and GSK3β, and Fzd1, 2, and 7. Direct binding of Fzds prevents binding of endogenous Wnt ligands and silences pathway activity. This promotes epithelium permeability and a pro-inflammatory state. **(B)** During *H. pylori* infection of gastric mucosa, CagA activates canonical Wnt signaling through the breakdown of adherens junctions, and non-canonical Wnt signaling through stimulation of CaN and NFAT nuclear translocation by an unknown mechanism. Canonical pathway signaling is also activated through unknown T4SS effectors that induce phosphorylation of LRP and DVL and inhibit GSK3β, promoting cytosolic accumulation of β-catenin and subsequent nuclear translocation to activate genes that drive cell proliferation. Furthermore, unknown mechanisms induce methylation of Wnt pathway inhibitory genes *SFRP1* and *DKK1* to hypothetically facilitate Wnt signaling activity during infection. **(C)** *P. aeruginosa* is decorated with lectin LecB which induces β-catenin degradation through an unknown extracellular mechanism, resulting in decreased cell proliferation. Coincidentally, NF-κB signaling is activated and inflammatory target genes *IL6, IL1B*, and *TNFA* are expressed. LecB as well as the quorum-sensing molecule PAI both induce disruption of adherens junction in infected epithelia, contributing to prolonged tissue damage in the host.

TcdA and TcdB exert enterotoxigenic effects including weakening of the epithelial barrier through disruption of tight junctions caused by the glucosylation of small GTPases that regulate actin filamentation, and stimulation of epithelial cells to recruit immune cells and program a pro-inflammatory response (110). Additionally, *C. difficile* is known to inhibit cell proliferation within the intestinal epithelium, a process typically regulated by canonical Wnt signaling, through the direct inhibitory effect of TcdA and TcdB on the Wnt pathway (Figure 6A) (114). TcdA has been shown to induce β-catenin degradation in the presence of pathway stimulation by Wnt3a, as well as prevent stimulation of pathway activity following pretreatment of intestinal epithelial cells with Wnt3a. Consequently, *CMYC* transcription is suppressed resulting in cell cycle arrest (42). TcdA-mediated degradation of β-catenin is independent of the β-catenin destruction complex, as TcdA stimulation of cells expressing a GSK3β phosphorylation-resistant β-catenin mutant cannot rescue pathway inhibition. It is speculated that TcdA glucosylation of Rac1 also inhibits canonical Wnt signaling as Rac1 expression is necessary for β-catenin nuclear translocation in certain cell types (115).

Recent studies have identified a unique role for TcdB in inhibition of Wnt signaling. Through sgRNA screening, TcdB has been shown to directly bind Fzd receptors in the intestinal epithelium, including Fzd1, Fzd2, and Fzd7 (43). This results in inhibition of LRP and Dvl phosphorylation, indicating a TcdB mechanism of Wnt inhibition through direct manipulation of the Wnt receptor complex. Wnt ligands interact with Fzd through two distinct binding sites, as well as hydrophobic interactions between a groove on the Fzd-CRD and the palmitoleic acid moiety (PAM) of Wnt (116). TcdB interacts with the Fzd-CRD as well, although the binding site is distinct from the Wnt binding sites as revealed by the co-crystallization of TcdB Fzd-binding domain and Fzd-CRD (44). In the pathway off state under normal physiological conditions, Fzd-CRD binds an endogenous lipid which becomes displaced by the Wnt PAM when the signal-initiating ligand binds. The endogenous lipid acts as coreceptor for TcdB that TcdB directly engages and locks in place, preventing displacement by the Wnt PAM and inhibiting pathway activation. It was also demonstrated that TcdB can bind Fzd when a Wnt ligand has already bound, with the Wnt PAM acting as a TcdB coreceptor, suggesting TcdB may have other inhibitory mechanisms including inhibition of receptor dimerization which has been shown to be stimulated through Wnt binding and necessary for signal transduction to occur (44, 117). This blockade of Wnt signaling is hypothesized to be a major underlying cause of weakening of the intestinal epithelium, tissue damage, and inflammation, hallmarks of PMC and *C. difficile* infection (44).

The unique inhibitory mechanisms of toxins TcdA and TcdB on Wnt signaling in host cells has expanded the model of pathogen manipulation of Wnt signaling through the identification of both intra- and extra-cellular molecular interactions that contribute to *C. difficile* pathogenesis. It remains to be discovered if TcdA glucosyltransferase activity modulates non-canonical Wnt signaling in the intestinal epithelium due to the major role of small GTPases in non-canonical pathways (118). Further investigation of TcdB interference with Fzd receptors will also lend insight into the critical interactions for Wnt pathway signaling initiation under normal physiological conditions. Of note, the canonical Wnt pathway components APC, GSK3β, Wnt5a, and LRP6 were also identified as potential targets of TcdB in an sgRNA screen (43). Whether the pool of TcdB that is endocytosed has a relevant role in Wnt signaling manipulation through interactions with these intracellular components remains to be determined. Of particular interest is the exploitation of TcdB-Fzd interaction for rational drug design. A recombinant TcdB mutant lacking Fzd-binding ability can induce significantly less swelling, epithelial damage, and infiltration of immune cells in a mouse cecum injection model, demonstrating that modulation of this toxin activity in the intestines has strong potential to reduce disease severity (44).

### Helicobacter pylori

*Helicobacter pylori* is a Gram-negative bacilli and most common infectious agent found in both adults and children worldwide. The bacterium is transmitted fecal-orally and oral-orally and replicates within the mucin layer in the stomach adhered to gastric epithelium. Pathogenic *H. pylori* strains possess the *cag* pathogenicity island, a genetic locus that consists of 31 genes including the effector CagA and the T4SS that secretes it. CagA is injected into gastric epithelial cells and directly interferes with cellular signaling pathways such as NF-κB and MAPK signaling, alters cell-cell contacts, and stimulates a proinflammatory response, thereby promoting the *H. pylori* replicative niche (119). CagA induction of the cell cycle and host survival pathways including canonical β-catenin-dependent Wnt signaling induce cellular transformation and replication which is why Cag^+^ strains of *H. pylori* increase host predisposition to tumorigenesis and gastric carcinoma, and *H. pylori* is classified as a type I carcinogen (120, 121).

While it is well-documented that *H. pylori* infection activates β-catenin-dependent Wnt signaling in the gastric epithelium, the role of CagA in Wnt activation is controversial. An early observation in the field determined that during infection of human gastric epithelia, β-catenin is stabilized. This was associated with infection by CagA^+^ strains (Figure 6B) (122). The mechanism for activation of β-catenin has been shown to be in part mediated by CagA disruption of adherens junctions. CagA directly interacts with E-cadherin which causes β-catenin dissociation from the adherens junctions complex and subsequent translocation to the nucleus (45). CagA induction of β-catenin target gene CDX and transactivation of p21 links disruption of adherens junctions and Wnt pathway activation with *H. pylori* CagA carcinogenesis as these gene products are required for transformation of gastric epithelium to intestinal epithelium which is a critical event in the development of intestinal metaplasia. An earlier finding connected activation of non-canonical NFAT-dependent Wnt signaling with CagA induction of p21, demonstrating regulation of Wnt signaling by CagA at multiple levels during infection (47). In a mammary epithelium model of infection, adherens junctions proteins were shown to dissociate upon infection in a CagA-independent mechanism resulting in loss of junctional integrity (123). While this group demonstrates β-catenin is stabilized, they do not detect nuclear localization of the transcription factor, but rather propose the hypothesis some other factor is able to cleave E-cadherin and manipulation junctions (123). This could indicate CagA^−^ strains of *H. pylori* stimulate β-catenin signaling through other mechanisms, or these results could suggest host factor-dependent predisposition to pathway hyperactivation.

Thus, a CagA-independent mechanism of Wnt activation may be active during *H. pylori* infection in addition to manipulation of the signaling pathway through interference with adherens junctions. LRP5/6 and Dvl are both phosphorylated in a Cag-independent, T4SS-dependent manner in a gastric epithelial infection model (124). *H. pylori* has also been shown to inhibit GSK3β activity which suppresses β-catenin degradation and is shown to induce Wnt pathway target gene *CCND1* (125, 126). Intracellularly, β-catenin activation and Wnt target gene expression is linked to activation of PI3K and Akt which inhibit GSK3β during infection, but the role of the Wnt receptor complex protein LRP6 in this mechanism remains to be investigated. Ultimately, there may be cell type- and temporally-specific deregulation of Wnt signaling during infection, as well as diverse effects of different effectors, that promote a tightly controlled differentiation vs. proliferation profile during *H. pylori* infection that ultimately serves to facilitate bacterial survival and dissemination.

*H. pylori* is well-known to modify target cell epigenetics which can also promote infection and tumorigenesis (127). A recent report linked *H. pylori* infection with modulation of Wnt signaling through epigenetic modification of Wnt pathway-related genes. Human *H. pylori*-positive gastric carcinoma samples display significantly increased methylation at the host genes *SFRP1* and *DKK1*, inhibitors of Wnt signaling (46). This corresponded with slight to moderate increases in β-catenin nuclear localization. Patient samples that demonstrated *H. pylori* eradication at a one-year follow-up did not have significantly altered epigenetic footprints, indicating *H. pylori* can induce long-lasting changes to host genomics and impact Wnt signaling activity in hosts post-eradication (46).

Understanding the connection between *H. pylori* secreted effectors and changes to host cell signaling pathways provides insight into potential avenues for therapeutics, infection or gastric cancer biomarkers, and pathogen-mediated tumorigenesis. Both CagA-dependent and -independent mechanisms are at play during infection to manipulate Wnt signaling and facilitate an environment within the host that supports bacterial replication. The role of LRP in activation of signaling and CagA cleavage of E-cadherin demonstrates *H. pylori* may utilize secreted proteins to manipulate Wnt signaling at the level of the receptor complex to induce pathway activity, a model of pathway activation applicable to intracellular and extracellular pathogens alike. Interestingly, pathways active during infection including PI3K-AKT and Wnt signaling both converge at the inactivation of GSK3β, indicating redundant mechanisms in place to ensure the different outcomes that are mediated by inhibition of the kinase. Further understanding of how the kinase activity of GSK3β is a crux for infection may lead to the development of therapeutics that act on cellular signaling pathways and have the potential to delay pathogen-induced tumorigenesis.

### Pseudomonas aeruginosa

*Pseudomonas aeruginosa* is a Gram-negative, opportunistic pathogen and a causative agent of microbial keratitis, burn wound infections, and hospital-acquired pneumonia. The bacteria is found in biofilms that are essential for host attachment and survival (128). *P. aeruginosa* utilizes quorum-sensing (QS) systems to sense bacterial population density and regulate biofilm development, mammalian host defense, host-microbe interactions, virulence and metabolite acquisition (129, 130) *P. aeruginosa* pathogenesis also encompasses disruption of epithelial barrier function, increased inflammatory response, and bacterial virulence factors.

Canonical Wnt signaling plays a major role in epithelial barrier integrity and proliferation during *P. aeruginosa* infection. Host response to *P. aeruginosa* infection leads to activation of the Wnt signaling pathway to regulate the pro-inflammatory response and promote bacterial clearance; therefore, inhibition of Wnt signaling is advantageous for persistent infection and survival. A recent study has shown that macrophages stimulated with Wnt3a conditioned media inhibits production of pro-inflammatory cytokines IL-6, IL-1β, MIP2, and TNF-α during *P. aeruginosa* infection. Wnt3a treatment also promotes apoptosis of macrophages at 12 and 24 hpi and enhances intracellular bacterial killing via upregulation of anti-microbial peptides CRAMP and BD1 (131). During *P. aeruginosa* infection inhibition of Wnt3a upregulates expression of pro-inflammatory cytokines, likely to delay tissue recovery and promote *P. aeruginosa* colonization (Figure 6C) (49). In a *P. aeruginosa* keratitis model of infection, melting of the cornea occurs due to bacterial proteases, activation of matrix metalloproteases, and deregulated immune response (132). β-catenin inhibits pro-inflammatory cytokines and decreases bacterial burden in the corneal stroma to reduce the severity of *P. aeruginosa* keratitis (133). Overexpression of β-catenin in B6 corneas results in suppression of *IL1B, MIP2*, and *TNFA* in phagocytes, but not in corneal epithelial cells, for host resistance of *P. aeruginosa* infection. Bacterial load is also decreased during overexpression of β-catenin in B6 corneas infected with *P. aeruginosa*. Collectively these studies suggest inhibition of Wnt signaling, specifically by downregulation of Wnt3a and β-catenin, increases bacterial burden and the inflammatory response during *P. aeruginosa* infection. Along with these findings, lithium chloride (LiCl) was shown to promote bacterial clearance. LiCl promotes canonical Wnt pathway activity through the inhibition of GSK3β (134). Various studies have determined that GSK3β is important in regulating inflammatory responses during bacterial infections (135). For example, *Klebsiella pneumoniae*-infected mice provided with LiCl-treated drinking water display increased survival and decreased liver injury due to a decrease in bacterial burden and cytokine production in blood and liver tissues (136). During *P. aeruginosa* infection, LiCl has been shown to inhibit proinflammatory cytokine TNF-α, enhance production of anti-inflammatory cytokine IL-10, and ultimately lead to host resistance against *P. aeruginosa* infection in the cornea (137). In correlation with previous listed studies, a reduction in inflammatory infiltration is observed in LiCl-treated cells, and increased apoptosis of phagocytes occurs. Taken together, *P. aeruginosa*-mediated inhibition of Wnt/β-catenin signaling is important for recruitment of infiltrating phagocytes and increased inflammatory response to promote dissemination and survival of the bacteria.

As previously described, β-catenin is a major contributor to the integrity of adherens junctions and may play an indirect role in the integrity of tight junctions and epithelial barrier during *P. aeruginosa* infection. *P. aeruginosa* contains two quorum-sensing systems which regulate specific signaling molecules that contribute to infection and survival (138). Adherens junctions integrity is decreased by a *P. aeruginosa* acyl-homoserine lactone (PAI) quorum-sensing molecule (48, 139). Treatment of CaCO-2 cells with the PAI results in a decrease in adherens junction-associated proteins E-cadherin and β-catenin expression during early infection. Hyperphosphorylation of occludin, ZO-1, E-cadherin and β-catenin are detected on tyrosine residues during early infection; however, occludin and β-catenin are dephosphorylated on serine and threonine residues. These outcomes are responsible for dissociation of E-cadherin-β-catenin complexes and association of ZO-1-occludin complexes ultimately leading to disassembly of adherens junctions (48). Of note, the expression levels of E-cadherin and β-catenin increase back to normal levels at later time points of infection (5–24 h), demonstrating a reversible effect of PAI during *P. aeruginosa* infection. Taken together, distinct phosphorylation events on adherens junction proteins, including β-catenin, play an important role in *P. aeruginosa* PAI-mediated tissue damage.

Along with *P. aeruginosa* PAI, soluble carbohydrate-binding lectin LecB is regulated by both *las* and *rhl* QS systems and reprograms the Wnt signaling pathway for infection and survival. LecB is one of two lectins expressed on the outer bacterial membrane of *P. aeruginosa* and is important for adhesion, biofilm formation, pilus biogenesis, and protease activity (140). LecB acts antagonistically to Wnt signaling through proteasomal degradation of β-catenin in a GSK3β-dependent manner, resulting in a G1-S phase cell cycle arrest in lung epithelial cells (49). LecB reduces β-catenin at adherens junctions and causes accumulation of the protein at centrosomes expressing high levels of proteasomes. Furthermore, co-incubation of Wnt3a and LecB repressed nuclear translocation of β-catenin, indicating infection actively suppresses canonical Wnt signaling activation. In addition, Ser536 phosphorylation of p65, a marker for increased transcriptional activity and acetylation of NF-κB, increases during *P. aeruginosa* infection, resulting in activation of NF-κB and subsequent elevation of downstream target genes *TNFA, IL6*, and *IL1B* which are linked to delayed tissue recovery (49). Taken together, these results indicate that *P. aeruginosa* manipulates the Wnt/β-catenin signaling pathway via β-catenin degradation and NF-κB activation to delay tissue recovery and promote infection.

*P. aeruginosa* is an opportunistic pathogen that utilizes various approaches to cross the epithelium at cell-cell junctions, including disruption of the epithelial cell barrier via PAI induction of phosphorylation of E-cadherin-β-catenin complexes (141). *P. aeruginosa* PAI also modulates mechanisms of immune tolerance to provide a niche for bacterial survival (142). Targeting the quorum sensing abilities of *P. aeruginosa* has been explored as an antimicrobial strategy, so understanding the extent of PAI in manipulating the host cell may identify mechanisms by which anti-quorum sensing drugs can also target mechanisms involving Wnt signaling. As previously described, *S. typhimurium* infection results in simultaneous β-catenin degradation and increased NF-κB activity in a murine model (18). *P. aeruginosa* LecB degrades β-catenin to decrease proliferation of lung epithelial cells, ultimately leading to delayed tissue recovery and persistent infection. Further investigation of the role of LecB-mediated β-catenin degradation inducing activation of NF-κB should also be explored. LecB is localized to the outer membrane of *P. aeruginosa* and binds to host cell plasma membrane receptors stimulating changes to epithelial barrier function and increased inflammatory response (143). Therefore, LecB-dependent β-catenin degradation may be stimulated by direct binding to extracellular portions of the Wnt receptor complex or extracellular adherens junction components. Understanding the mechanisms utilized by LecB to manipulate Wnt signaling to decrease proliferation and tissue recovery of lung epithelial cells may serve as a potential therapeutic approach to *P. aeruginosa* infection.

### Escherichia coli

Uropathogenic *Escherichia coli* (UPEC) is an extraintestinal pathogenic isolate of *Escherichia coli* and the causative agent of 40% of hospital- and 80% of community-acquired urinary tract infections (144). UPEC infects the urothelium, a highly specialized epithelium lining the lower urinary tract that is composed of basal, intermediate, and superficial cell layers (145). The superficial apical cell layer provides the primary urinary barrier and is composed of large, hexagonal cells known as umbrella cells which are exfoliated and regenerated upon infection (145, 146). Upon infection, UPEC enters and ascends into the urinary tract, enters the urethra, and migrates into the bladder lumen where it engages with bladder umbrella cells by binding to mannose-containing glycoprotein receptors via the adhesin FimH (50). Engulfment into the host cell is followed by the formation of intracellular bacterial communities (IBCs) that demonstrate biofilm-like properties (147, 148). UPEC detach from the intracellular biofilm and establish intracellular colonization leading to eruption of bladder epithelial cells. Dissemination and detachment lead to UPEC mobilization to the bladder lumen which increases the risk of bacteremia and septicemia (148).

UPEC has evolved several molecular-based strategies to subvert the innate immune response for efficient colonization and persistent infection in the urinary tract. One mechanism of host response to UPEC infection is the process of exfoliation of bladder epithelial cells that are overwhelmed with bacteria (146, 147). To renew exfoliated superficial epithelium, genetic programs are activated for proliferation and differentiation of urothelial cells (149). Despite the role of exfoliation in bacterial clearance, this outcome also leads to exposure of immature bladder epithelial cells which are more susceptible to UPEC infection (50). Within these compartments, bacteria are in a quiescent state; however, as the immature urothelial cells differentiate into mature urothelial cells, UPEC replicates for recurrent infection (147). Modulation of Wnt signaling has been linked to differentiation and proliferation of basal/intermediate cells during UPEC infection. More specifically, expression of Wnt5a during UPEC infection leads to proliferation of basal/intermediate cells while suppression of Wnt5a expression results in differentiation (50). A recent study demonstrated suppression of Wnt5a/Ca^2+^ signaling promotes differentiation of basal/intermediate cells prior to exfoliation (50). Infection by FimH^+^ UPEC results in a decrease in Wnt5a expression and subsequent decrease of non-canonical Wnt pathway targets PKCδ and CamKIIδ up to relatively late infection timepoints (50). This suggests that prior to exfoliation, non-canonical Wnt signaling is suppressed to promote differentiation of umbrella cells upon initiation of UPEC infection. However, it has also been shown that UPEC induces infected cells to secrete paracrine factors that cause alterations in the expression of the epigenetic writer EZH2 which enhances Wnt5a expression, promoting proliferation of infected cells at relatively early infection time points (51). Further studies must be done to determine if there is temporal regulation of Wnt5a expression during UPEC infection. Despite these discrepancies, research suggests that modulation of Wnt signaling during UPEC infection promotes exfoliation and cell proliferation for dissemination and survival, respectively. Consistent with these findings, EZH2 has been shown to be involved in Wnt-mediated proliferation in *Citrobacter rodentium* infection (52). *C. rodentium* is a Gram-negative, murine enteric bacterial pathogen and closely related to enteropathogenic (EPEC) and enterohaemorrhagic *E. coli* (EHEC) (150–152). EPEC, EHEC, and *C. rodentium* are members of the attaching and effacing (A/E) family of bacterial pathogens due to the destructive effect of colonization of the intestinal epithelium and enterocytes (150, 151). The natural host range and genetic make-up of *C. rodentium* makes it a good *in vivo* model for A/E pathogens. EZH2 represses Wnt inhibitory factor WIF1 resulting in activation of Wnt/β-catenin signaling and ultimately *C. rodentium*-induced crypt hyperplasia and tumorigenesis (52). Therefore, modulation of the Wnt signaling pathway by A/E pathogens would appear to be a useful regulatory mechanism by providing a niche for adaptation and survival in the host.

Along with a diverse range of adhesin molecules, UPEC utilizes a variety of toxins to increase invasiveness and facilitate virulence. Of these secreted toxins is a pore-forming, T1SS toxin α-hemolysin (HlyA). HlyA is inserted into epithelial and macrophage membranes and triggers rapid degradation of various host proteins involved in the proinflammatory response and cell-cell and cell-matrix interactions, including β-catenin (153). HlyA mediated degradation of β-catenin in infected BECs occurs simultaneously with loss of IκBα. Additionally, the NF-κB subunit RelA (p65) is also degraded in HlyA-intoxicated bladder epithelial cells. HlyA-mediated degradation of RelA (p65) correlates with reduced *IL6* expression (153). This study suggests a novel mechanism by which UPEC inhibits NF-κB-mediated inflammatory response via β-catenin degradation, and independent of IκBα-mediated proteasomal degradation. Similar results were shown with macrophages, demonstrating a significant role of HlyA in modulating phagocytes and epithelial cell function for UPEC survival. Investigation of the cross-regulation of β-catenin and NF-κB signaling pathways during UPEC infection would demonstrate if UPEC utilizes Wnt signaling as a means of immunosuppression.

Several gaps in knowledge remain in reference to the role of Wnt signaling in subversion of innate immunity during UPEC infection. While exfoliation and proliferation are mechanisms of host response to UPEC infection, this response is beneficial for establishing a niche for persistent infection (153, 154). Identifying the role of Wnt signaling and possible UPEC effectors involved in pathway activation to induce exfoliation and proliferation for survival may allow for better understanding of how UPEC infections can persist in the face of host defenses. Inhibition of Wnt5a-induced non-canonical signaling and EZH2 may potentially inhibit proliferative processes necessary for UPEC infection and may therefore serve as potential therapeutic targets.

## Concluding Remarks—Winning the Tug-of-Wnt

Pathogens' respective niches and modes of dissemination shape how they avoid innate immune pressure during infection. Wnt signaling is a host cell pathway targeted by both obligately intracellular and extracellular bacterial pathogens alike, demonstrating the breadth of the pathway's control over eukaryotic cellular processes. A consistent theme among the intracellular pathogens discussed in this review is activation of the pathway in the host cell. Evidence from *Salmonella, Chlamydia, Rickettsia*, and *Mycobacteria* demonstrates canonical pathway activation contributes to an anti-inflammatory state which mediates immunosuppression and may prolong infection. The former two pathogens also appear to stimulate canonical Wnt signaling to prolong host cell survival by inducing either proliferation or differentiation of the host cell. *Ehrlichia* is unique in that studies have shown the bacterium activates non-canonical pathways which govern cytoskeletal reorganization and autophagy regulation, permitting phagocytosis of the bacterium without destruction by the lysosome. Inhibition of the canonical Wnt pathway by *Salmonella* and *C. difficile* occurs in intestinal epithelium and the intestinal capillary endothelium for the former and in the colonic epithelium for the latter, and contributes to pathogen manipulation of tissue barriers, facilitating dissemination into the bloodstream. In the case of *Salmonella*, inhibition of Wnt signaling may also lead to an inflammatory response that recruits target cells for Salmonella and promotes dissemination. Studies have shown that *C. difficile* and *P. aeruginosa* utilize multiple effector molecules to interfere with canonical Wnt signaling activity of the surrounding tissue during infection. In both cases, viability of the cell within the infected tissue is reduced which may contribute to nutrient accumulation or dissemination for the bacteria. *E. coli* and *H. pylori*, contrastingly, activate canonical and non-canonical Wnt signaling from their extracellular niches, resulting in increased proliferation or differentiation of cells in the surrounding epithelium. Although evidence is yet to demonstrate a clear phenotype, it is likely that pathway activation also contributes to an anti-inflammatory state in the bacterial replicative niche due to reciprocal regulation of the transcription factors NF-κB and β-catenin.

Studies have implicated a range of bacterial effectors with the capability to manipulate canonical and non-canonical Wnt signaling, including T1,3,4SS effector proteins of *S. enterica, C. pneumoniae, E. chaffeensis, E. coli*, and *H. pylori*; lectins and quorum-sensing molecules of *P. aeruginosa*; two-component regulatory systems of *S. enterica*; and likely many more unknown factors. Shared mechanisms involve disassembly of adherens junctions as is the case for *C. trachomatis, R. rickettsii, H. pylori*, and *P. aeruginosa* which may be a means for increasing the amount of cytosolic β-catenin available to activate the Wnt response element, or a means of inactivating the protein and facilitating its degradation. Cpn1027 of *C. pneumoniae* and TcdB of *C. difficile* both inactivate pathway components through direct binding. Such may be the case for *E. chaffeensis* TRP32, 47, and 120, as many host-bacterial interactions between negative regulators of Wnt signaling and the TRPs have been identified for which mechanisms have yet to be described. Pathogens also deploy enzymes during infection to manipulate Wnt signaling components at the post-translation modification level, as is the case with *C. difficile* TcdA and *S. enterica* AvrA and SopB. Research identifying Wnt pathway regulatory mechanisms and foreign molecules that can interfere with the pathway has applications ranging from research tools to potential therapeutics.

As research detailing mechanisms of pathogenesis and bacteria-host interaction accumulates, our knowledge of how various cellular mechanisms participate in innate immune signaling expands. Wnt signaling regulates stem cell renewal, cell proliferation, and cellular morphology, but the ability of pathogens to usurp these processes to establish infection demonstrates that Wnt signaling is also a target for bacterial immunoevasion strategies. By drawing a parallel between pathway dysregulation during infection and dysregulation in “classical” diseases of Wnt signaling, we can identify targetable pathway components for drug intervention of infectious disease. One such strategy involves the use of the existing armaments of small molecule inhibitors and activators that target various components of canonical Wnt signaling thereby overriding the pathogen reprogramming strategy. Another approach is the use of antivirulence therapy to target the pathogen-host molecular interactions that are beneficial to bacterial virulence, thereby weakening the pathogen through impairing its ability to manipulate the Wnt pathway.

Multiple small molecules have been identified that modulate Wnt signaling activity through activation or inhibition of pathway components or regulators (14). Use of these drugs as an approach for infection control is most suitable for bacteria for which stimulation or repression of pathway activity significantly impairs establishment of infection or a damaging inflammatory response. GSK3 promotes Wnt signaling through inactivation of the destruction complex and a number of small molecules target GSK3 activity. These inhibitors have been used in mouse infection models for *Streptococcus* and *Porphyromonas gingivalis* and demonstrate efficacy in reducing bacterial burden or excessive inflammation (155–157). GSK3 represents an attractive target for drug design because of its suppressive effect on NF-κB signaling, and upregulation of its kinase activity by different pathogens makes it a potential point for therapeutic intervention during infection (158). Modulators of extracellular components of Wnt signaling include pathway agonists that mimic Wnt ligands and neutralizing antibodies that target secreted pathway inhibitors (159–161). These types of drugs may be utilized when infection causes suppression of Wnt signaling which leads to tissue damage. *Salmonella* perturbation of the GVB represents a case in point—stimulation of Wnt signaling reverses infection-induced endothelial weakening (20). The effectiveness of these potential Wnt-modulating therapeutics will be impacted by the particular points within the Wnt signal cascade that different pathogens exert manipulation, which prospective mechanism-oriented studies should address. Ultimately, the vast amount of signaling proteins within the Wnt pathway suggests that while this cellular signal cascade can be targeted by drugs, such intervention should be thoroughly researched to anticipate off-target and adverse side effects (162).

Studies of how pathogens interact with the host signalosome allows us to exploit this knowledge to target crucial interactions between bacterial and host factors that enable bacterial virulence (163). Antivirulence therapy has risen as a strategy to combat pathogenic bacteria by disarming them of virulence factors, and an in-depth understanding of the interplay between virulence factors and Wnt signaling enables the development of such therapeutic strategies. For example, *C. difficile* infection has been shown to be controlled by small molecules and monoclonal antibodies that target critical enzymatic or protein-protein interaction-mediating domains within TcdB (164, 165). The identification of a Fzd-binding domain within TcdB uncovers a target against which small molecules or neutralizing antibodies can be directed, thereby reducing the pathological effects of bacterial inhibition of Wnt signaling within the intestines. A similar strategy could be directed toward *E. chaffeensis* infection, as surface TRPs including TRP120 mediate phagocytosis of the bacterium through stimulation of Wnt pathway activity. As further research identifies the molecular determinants of such pathological events, interference with TRP-Wnt receptor complex could reduce the ability of *E. chaffeensis* to establish infection within monocytes. *P. aeruginosa* utilizes surface protein LecB to extracellularly modulate β-catenin activity. Antivirulence drugs in preclinical development that target LecB demonstrate how this protein can be manipulated to reduce infection, and suggest that the efficacy of these drugs is, at least in part, due to the disruption of *P. aeruginosa* modulation of Wnt signaling (166).

The era of Wnt signaling research has shifted from understanding the basic molecular biology of the pathway to identifying how the pathway is manipulated by infectious agents. The ultimate goal is to use this knowledge to repurpose or develop novel therapeutics for infectious disease that are relevant in the age of antibiotic resistance. Pathogens have coevolved with the human host and are master manipulators of signaling pathways that have been in place throughout the evolution of metazoans. Understanding the interactions that dictate how pathogens can usurp the Wnt signal cascade to benefit their survival and dissemination facilitates the development of counter-defense strategies that enable the host innate immune system to control and clear infection.

## Author Contributions

MR and JM conceived the work. MR gathered information and contributed all sections except for *P. aeruginosa* and *E. coli* which were contributed by LP. JW performed artwork. All authors participated in editing of the final draft.

### Conflict of Interest

The authors declare that the research was conducted in the absence of any commercial or financial relationships that could be construed as a potential conflict of interest.
